# Randomized controlled trial of a minimal versus extended Internet-based intervention for problem drinkers: study protocol

**DOI:** 10.1186/s12889-015-1347-8

**Published:** 2015-01-21

**Authors:** John A Cunningham, Christian S Hendershot, Jürgen Rehm

**Affiliations:** Department of Social and Epidemiological Research, Centre for Addiction and Mental Health, Toronto, Canada; National Institute for Mental Health Research, Australian National University, Canberra, Australia; Campbell Family Mental Health Research Institute, Centre for Addiction and Mental Health, Toronto, Canada; Department of Psychiatry, University of Toronto, Toronto, Canada; Dalla Lana School of Population Health, University of Toronto, Toronto, Canada; Technische Universität, Dresden, Germany

## Abstract

**Background:**

Problem drinking causes great harm to the person and to society. Most problem drinkers will never seek treatment. The current trial will test the efficacy of two Internet interventions for problem drinking – one minimal and the other extended – as an alternate means of providing help to those in need.

**Methods/Design:**

A double blinded, four-wave panel design with random assignment to two experimental conditions will be used in this study. Participants will be recruited through a comprehensive recruitment strategy consisting of online and print advertisements asking for people who are ‘interested in helping us develop and evaluate Internet-based interventions for problem drinkers.’ Potential participants will be screened to select problem drinkers who have home access to the Internet. Participants will be sent to a password-protected Internet site and, upon signing in, will be randomized to be provided access to the minimal or extended Internet-based intervention. Six-month, twelve-month, and two-year drinking outcomes will be compared between experimental conditions. The primary hypothesis is that participants in the extended Internet intervention condition will display significantly improved drinking outcomes at twelve months compared to participants in the minimal intervention.

**Discussion:**

The findings of this trial will contribute to the growing literature on Internet interventions for problem drinkers. In addition, findings from this trial will contribute to the scarce literature available evaluating the long-term efficacy of brief interventions for alcohol problems.

**Trial registration:**

Clinical Trials.gov #NCT01874509; First submitted June 17, 2013.

## Background

Hazardous alcohol use is the second leading contributor to the preventable burden of disease in high income countries [1-4]. In 2001, 6% of all deaths in Canadian adults under the age of 70 were attributable to alcohol [3]. In addition, the economic costs of problem drinking are also high. In 2002, it was estimated that alcohol-related health care cost Canadians $2.3 billion dollars [5] and there are also other substantial economic costs, mainly attributed to lost productivity and law enforcement [4]. Alcohol use is thus a key factor in population health in Canada.

There are many more problem drinkers than those with alcohol dependence [6]. The majority of these problem drinkers will never seek treatment [7-10]. Nevertheless, studies assessing the level of interest in self-help materials for problem drinkers in the general population reveal that a considerable number of drinkers, especially heavier drinkers, would like to receive aids to help them drink less [11]. Koski-Jännes and Cunningham [12] found that 39% of current drinkers were interested in computerized summaries comparing their drinking to that of other Canadians, and that fully 70% of problem drinkers were interested in receiving such a self-help intervention.

The widespread accessibility of the Internet may prove it an ideal vehicle for the delivery of self-help material to problem drinkers. A recent systematic review [13] emphasized the fast growing body of evidence for the evidence supporting Internet-based Interventions (IBIs). The majority of this research suggests that personalized feedback interventions can reduce drinking over short intervals [14-17]. However, there is little research evaluating the efficacy of more comprehensive web-based interventions for facilitating long-term changes in drinking [18]. This limitation is notable for at least two reasons. First, initial changes in alcohol use are most often not sustained [19,20]; and the strategies needed to maintain long-term change may be different than those required for initial change [21-23]. Whereas personalized feedback could provide a source of initial motivation to reduce drinking, more comprehensive cognitive-behavioural strategies may be more effective for aiding in sustained behaviour change and relapse prevention. Second, there have been increasing calls to adapt efficacious alcohol treatments to allow for sustained delivery, consistent with a continuing care model of treatment [24-26]. One advantage of IBIs is that they can be sustained indefinitely (i.e., the cost of long-term or comprehensive interventions will often be comparable to those of brief interventions).

### Pilot study

A pilot trial conducted by the authors provides preliminary evidence suggesting that IBIs that incorporate cognitive behavioural strategies may be more effective than those that provide personalized normative feedback. The pilot trial specifically assessed whether six-month outcomes varied between problem drinkers provided with minimal IBIs (i.e. brief normative personalized feedback) versus extended IBIs that include comprehensive cognitive-behavioural strategies. In this trial, participants were randomized into either an extended intervention (i.e. Alcohol Help Centre: AHC) or a minimal intervention (i.e. Check Your Drinking: CYD). The AHC is a website that contains a series of cognitive-behavioural and relapse prevention tools that have been found to be effective in promoting reductions in drinking in clinical settings, whereas the CYD is a brief screener designed to provide personalized normative feedback aimed at motivating reductions in drinking. Some evidence was found for the added impact of the AHC extended intervention above that observed on the CYD minimal intervention [27]. There were, however, several limitations of this pilot trial that will be addressed in the present study. First, the present study will employ a double blind design to protect against other sources of bias. Second, a long-term follow-up will be included to test the hypothesis that intervention-related reductions in drinking are sustained to a greater extent in the context of a more comprehensive intervention.

### Hypotheses

#### Primary hypothesis

Participants in the extended Internet intervention condition will display significantly improved drinking outcomes at twelve months compared to participants in the minimal Internet intervention condition.

Secondary Hypotheses:

- **Secondary Hypothesis 1**: Participants in the extended Internet intervention condition will display significantly improved drinking outcomes at six months compared to participants in the minimal Internet intervention condition.
- **Secondary Hypothesis 2**: Participants in the extended Internet intervention condition will display significantly improved drinking outcomes at two years compared to participants in the minimal Internet intervention condition.
- **Secondary Hypothesis 3**: Participants in the extended Internet intervention conditions will display significantly improved health-related quality of life at twelve months compared to participants in the minimal Internet intervention condition.
- **Secondary Hypothesis 4**: Participants in the extended Internet intervention condition who have more involvement with the AHC intervention between baseline and twelve-month follow-up will demonstrate more improvement in drinking outcomes at twelve-month follow-up, compared to respondents in the extended Internet intervention condition who have less involvement with the AHC intervention.

## Methods/Design

### Design

**Figure 1:**
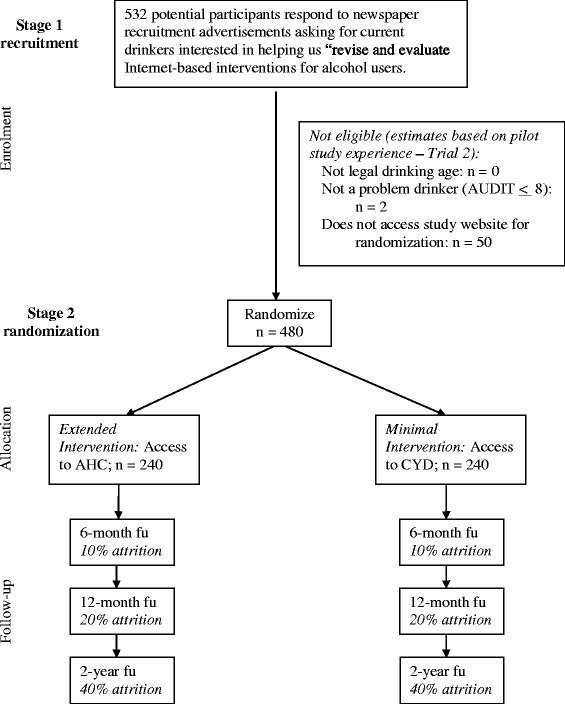
**Overview of the proposed intervention trial.**

This is a double-blind, four-wave panel randomized control trial of two IBIs for alcohol problems, with three follow-up periods (6 months, 12 months and 2 years). A comprehensive recruitment strategy using online and print advertisements will be used to recruit current drinkers interested in helping researchers “revise and evaluate Internet-based interventions for alcohol users.” Interested potential participants will be directed to complete an online consent form and a baseline questionnaire. Those found to be eligible based on their responses to the baseline questionnaire will be provided with a unique password to the study website. Upon accessing the website, they will be randomized into one of the two interventions: the minimal Internet-based Check Your Drinking personalized feedback intervention or the extended Internet-based Alcohol Help Center. Only participants who use their password and access the website will be included in the trial. Participants will be provided an honorarium of $20 for completing each follow-up questionnaire and an honorarium of $10 for initially accessing the study website (the latter will be employed in order to avoid loss of potential participants at this point). Potential participants deemed ineligible to participate will be compensated $20 for having completed the baseline questionnaire. See Figure 1 for a Consort Diagram summarizing this trial design.

### Ethical approval

The research methods to be used in this study have been approved by the standing ethics review committee of the Centre for Addiction and Mental Health.

### Participants – inclusion and exclusion criteria

Participation in the trial will be restricted to those who are 19 and above (legal drinking age in Canada), current problem drinkers (as indicated by a score of 8 or more on the Alcohol Use Disorder Identification Test - AUDIT) [28,29], have home access to the Internet and read English (the AHC is currently only available in English). It should be noted that this recruitment procedure may result in the participation of some individuals who are dependent on alcohol. However, as Heather p. 366, [30] has well summarized, “evidence shows that brief interventions are effective and should be used for individuals who are not actively seeking help at specialist agencies. This justification is again independent of level of seriousness, although most recipients of community-based interventions will obviously have problems of a less severe variety.” Indeed, as respondents in this trial will not be treatment seekers, the IBIs could easily serve as their first exposure to any services for alcohol problems. Thus, it is appropriate to include all eligible individuals in this evaluation, no matter the severity of their problems. Further it is also possible that some respondents will have accessed addictions treatment or other forms of assistance for alcohol problems (such as Internet-based interventions) at some point in their lives. This will be assessed on the baseline questionnaire, however respondents will not be excluded from the study due to prior treatment access because the intent is to evaluate the impact of the interventions in the extended range of potential community participants. However, random assignment to condition should ensure that socio-demographic characteristics, such as treatment access, will be randomly assigned across conditions.

### Interventions

#### Minimal intervention experimental condition

A randomized half of the subjects meeting eligibility criteria will be assigned to the Check Your Drinking screener (CYD; available at http://www.CheckYourDrinking.net). The CYD screener consists of an 18 item screener, the responses from which are then used to generate a personalized feedback Final Report containing normative feedback content as well as a summary of the amount and risks associated with the recipient’s drinking [31,32].

#### Extended internet-based intervention experimental condition

Subjects randomly assigned to the extended internet-based intervention will be directed to the Alcohol Help Centre (AHC; located at http://www.AlcoholHelpCentre.net). The AHC contains cognitive behavioral, motivational and relapse prevention components. In addition, there is an online support group moderated by health educators [33]. More details of the AHC are provided elsewhere [27].

### Randomization

After providing electronic consent, a letter will be sent to each participant, thanking them for agreeing to participate in the study. Contained in this letter will be a World Wide Web address unique to the study and a password unique to each participant. Upon accessing the website, the participant will enter his or her password and be randomly assigned to either the minimal or the extended Internet-based intervention using a simple randomization without replacement built into the website (no stratification or minimization given the large sample size in this trial).

### Blinding

Participants will be blind to their experimental condition as their password will always direct them to the same intervention after the initial randomization. Research staff involved in the trial will not be informed of respondents’ group allocation during interventions or at follow-up. The generation of the randomized sequence and loading of it into the server database has been conducted by a staff member who will not be involved in the implementation of the trial or with any participant contact.

### Outcome measures

#### Primary outcome measure

In order to gain a clear picture of changes in drinking status, both the quantity and frequency of alcohol consumption will be measured as well as the frequency of heavy drinking occasions [34,35]. Therefore, the primary outcome measure will be the AUDIT-C scale (composite measure of frequency of drinking, typical quantity of drinking on one occasion, and frequency of drinking five of more drinks on one occasion). This measure was chosen because it comprises the main predictors of risky drinking in a recent analysis of the relation of drinking to the development of alcohol dependence [36]. This measure was chosen rather than a drinking diary data collection method because summary measures generated from a drinking diary method have been shown to be functionally identical to single point retrospective measures [37] and because the inclusion of a drinking diary in research has been shown to increase attrition in studies employing mailed surveys [38].

#### Secondary outcome measure

Secondary outcome measures are: (1) number of drinks in a typical week [39-41]; and (2) highest number of drinks on a single occasion. Finally, Health related Quality of Live (HRQOL) will be measured at each time point using the WHOQoL-8. This short form has been used in a number of countries, is robust psychometrically, and overall performance is strongly correlated with scores from the original WHOQoL [42].

### Data analysis

#### Power analysis

The AUDIT-C measure is employed in this power calculation because this scale is a well-validated screener that has research supporting the amount of change that is needed in order to be clinically significant. Ideally, the study should be powered to be able to detect a difference in 1 point on the AUDIT-C scale as this was the added impact of the extended intervention over the minimal intervention observed in a previous pilot study (albeit at a six-month follow-up). A reduction from 9 points to 8 points on the AUDIT-C scale is also a clinically significant effect, representing 15% fewer people drinking beyond recommended low risk guidelines and a five drink per week reduction in typical weekly consumption (findings from the 2009 CAMH Monitor, a general population survey of Ontario residents) [43]. Further, a relatively variable sample is assumed with a standard deviation of 3.0 units on the AUDIT-C (standard deviation in the pilot trial was 2.5).

Following the convention that studies should be designed to have a statistical power of at least 80%, and that hypotheses be tested at the .05 level of significance, these specifications resulted in a final sample (required after attrition) of N = 286 (143 participants per condition).

In addition, there is allowance for the possibility of a substantial loss to follow-up in order to ensure sufficient numbers for analyses. Further, while the power analysis was conducted referring to the primary hypothesis (12 months follow up), sufficient participants will be recruited in order to test for a similar effect size at the two-year time point. Specifically, there is an allowance for a 20% attrition at the one-year follow-up and 40% attrition at the time of the two-year follow-up, and proposing to recruit a total of 480 participants at baseline (thus, allowing for a possible 40% loss to follow-up at the two year time point).

#### Analysis plan: primary hypothesis testing

The primary hypothesis is that participants in the extended Internet intervention condition will display significantly improved drinking outcomes at twelve months compared to participants in the minimal Internet intervention condition. This will be tested for the primary outcome variable, the AUDIT-C, using an analysis of covariance with experimental condition as the between subject variable and the baseline value of the outcome variable as the covariate. A maximum likelihood approach will be used to replace any missing data at twelve-month follow-up. Sensitivity analyses will be conducted with participants lost to follow-up excluded.

#### Secondary analyses

To test the difference across time between the two conditions (minimal or extended Internet-based intervention), a group (1, 2) x time (0, 6, 12, and 24) repeated measures MANOVA will be performed, including the primary outcome variable (AUDIT-C) and the two secondary outcome drinking variables (number of drinks in a typical week and greatest number of drinks on one occasion). Missing data will be replaced using a maximum likelihood approach. Planned contrasts will determine the nature of the differences observed. These planned contrasts will be stepped such that significant differences must be observed at the six months’ time point before comparisons will be made between condition at the twelve months’ time point, etc. Effect sizes will be calculated to determine the magnitude of differences in standard units. Interaction terms will be added to the MANOVA to test for sex differences (i.e., is there an interaction effect of subjects’ sex by experimental condition on drinking outcomes?). Finally, a separate repeated-measure ANOVA will be employed to test the hypothesis on the impact of receiving access to the Internet-based interventions on Health Related Quality of Life (secondary hypothesis 3). As part of these secondary analyses, we will also conduct a chi-square test to explore whether there is differential loss to follow-up between experimental conditions.

The proportion of each group that has sought further treatment (total and categorized into type of treatment such as support groups, residential, individual counseling) will be compared using χ^2^ statistics. If the proportion of treatment-seekers is high then further analyses will be conducted to explore the significance of this treatment to outcome.

## Discussion

The efficacy of IBIs to reduce drinking over short intervals has received increasing empirical evidence as is indicated in several systematic reviews [13-17]. However, research demonstrating that short-term changes in alcohol consumption are often not maintained [19,20] warrants an investigation of the efficacy of IBIs in long-term reductions in alcohol consumption. This project will be one of the first such trial to investigate the long-term benefits of IBIs among problem drinkers and compare the effectiveness of a normative personalized feedback intervention to one that contains a range of cognitive-behavioural and relapse prevention tools.
